# A Surface Thermal Sensing Framework for Internal Winding Temperature Estimation in Oil-Immersed Converter Transformers

**DOI:** 10.3390/s26144425

**Published:** 2026-07-12

**Authors:** Sheng Han, Zhiqiang Wang, Yuchen Tang, Li Huang, Hui Jiang, Xing Li

**Affiliations:** 1 Key Laboratory of Cleaner Intelligent Control on Coal & Electricity, Taiyuan University of Technology, Taiyuan 030600, China; hansheng@tyut.edu.cn (S.H.); zhiqiangwang_tyut@yeah.net (Z.W.); 2 College of Automation Engineering, Nanjing University of Aeronautics and Astronautics, Nanjing 211100, China; tangyuchen@nuaa.edu.cn; 3 China Electric Power Research Institute, Haidian District, Beijing 100192, China; 4 State Key Laboratory of Power Transmission Equipment Technology, Chongqing University, Chongqing 400044, China; jianghui@cqu.edu.cn

**Keywords:** oil-immersed transformer, internal winding temperature, prediction model, XGBoost-LSTM algorithm

## Abstract

Accurate monitoring of internal winding temperature is essential for assessing the thermal state and operational reliability of oil-immersed transformers. However, direct deployment of distributed temperature sensors inside transformer windings is difficult because of insulation constraints, structural complexity, and potential reliability risks. To address this problem, this paper proposes a non-invasive internal winding temperature estimation method based on surface temperature sensing and a hybrid deep learning model. In the proposed framework, external surface temperature measurements are used as sensor inputs to infer the internal transient thermal state of the transformer. First, an extreme gradient boosting (XGBoost) model is employed to evaluate the contribution of different surface temperature measurement points and select the sensing locations that are most strongly correlated with internal winding temperature variations. Then, the selected surface temperature time-series data are used to train a Long Short-Term Memory (LSTM) network, which captures the temporal evolution of the transformer temperature field under different operating conditions. The proposed method is verified through both numerical simulation and experimental testing on a scaled single-phase oil-immersed converter transformer model (D-800/35) developed in this study. The results show that the proposed XGBoost-LSTM model can estimate internal winding temperature with an error of less than 1.5 K. Compared with direct internal sensing, the proposed method provides a non-invasive and sensor-efficient solution for internal temperature monitoring. The results demonstrate its potential for real-time thermal state estimation, condition monitoring, and fault diagnosis of oil-immersed converter transformers.

## 1. Introduction

With the rapid development of the power industry, the demands for power system safety and supply reliability have steadily increased. As a critical equipment of power systems, the operational status of transformers is directly linked to the overall safety and stability of the system. Real-time monitoring of transformer health through various physical sensing technologies is a key strategy for ensuring secure and reliable power system operation. Among these, transformer temperature monitoring plays a vital role in assessing and preventing thermal damage, providing an essential safeguard for the safe and stable performance of the power system.

The internal winding temperature of a transformer is a key indicator of insulation faults and load fluctuations, making it highly significant for assessing the transformer’s overall health status. Currently, transformer winding temperature monitoring technologies can be broadly classified into two categories: direct measurement and indirect estimation. The direct measurement method involves installing temperature sensors, such as infrared sensors [1,2] and thermocouples [3], in the target area to collect temperature data and monitor transformer temperature rise. However, in practical engineering applications, embedding sensors inside the transformer is often challenging and may negatively impact the transformer’s structural integrity [4].

Indirect computation methods primarily include empirical formula approaches [5,6], thermal circuit models [7,8,9,10], and numerical algorithms. For instance, IEEE Standard C57.91-2025 [5] and IEC 60076-7 [6] provide empirical formulas for calculating the hotspot temperature of transformer windings. The thermal circuit model constructs a lumped-parameter thermal network for estimating the temperature at key internal nodes. Although empirical formula approaches and thermal circuit methods offer some engineering applicability, their accuracy is often limited, particularly under complex or varying operational conditions. Numerical algorithms represent another important approach for estimating transformer temperature, as they directly solve the partial differential equations governing the temperature field to obtain detailed temperature distributions, most notably through finite element method (FEM) simulations [11,12,13]. These simulations can yield highly accurate results by incorporating high-precision geometric models and accounting for the coupled effects of multiple physical fields [14,15]. However, such methods typically suffer from high computational costs and limited real-time performance, which restrict their practicality in dynamic monitoring applications.

In addition to the above methods, with the rapid advancement of artificial intelligence, artificial neural network (ANN) algorithms and heuristic approaches have been increasingly applied to address nonlinear problems in transformer temperature field modeling. Several researchers have conducted extensive studies in this area. As early as 2000, researchers explored the feasibility of using various neural network architectures to predict transformer top-oil temperature [16]. References [17,18] proposed a linear neural network trained using multidimensional parameters, including electrical, geometric, and environmental variables, to predict internal temperature states. In [19,20], time series models were developed to account for time-varying operational factors, leading to improved temperature prediction accuracy. Given the diversity and complexity of feature variables involved in ANN training [21], feature selection algorithms have been introduced in [22,23] to identify the most relevant inputs, thereby enhancing training efficiency. However, these approaches often involve a trade-off between model accuracy and computational efficiency. Overall, while ANN-based models exhibit promising reliability, particularly in time series prediction, they face challenges such as high demands for large, high-quality datasets and the difficulty of obtaining certain feature factors that can significantly impact model performance.

To address the challenges of difficult direct measurement of transformer internal temperature and low real-time performance of finite-element high-precision model temperature simulation, this paper proposes an XGBoost-LSTM prediction model for inferring internal temperature from transformer surface temperature. XGBoost is employed for efficient and effective feature selection, identifying the most relevant surface temperature points that correlate strongly with internal winding temperature. These selected features are then used as input to train an LSTM model, which excels at capturing temporal dependencies in time-series data. By integrating these two models, the proposed XGBoost-LSTM framework achieves both high prediction accuracy and improved computational efficiency. This method enables real-time, non-invasive estimation of internal transformer temperatures based on surface temperature measurements, offering a potential practical solution for transformer temperature status monitoring and fault prevention.

## 2. Thermal–Fluid Field Simulation of Power Transform

### 2.1. Physical Theory of Thermal–Fluid Field

During transformer working status, the current flowing through the windings generates a magnetic flux within the iron core, enabling energy transfer. Heat generation within the transformer primarily arises from two sources: Joule heating in the windings, known as copper loss, and eddy current and hysteresis losses in the core, collectively referred to as core loss. These heat sources contribute to the temperature rise within the transformer. The governing equations [24] for the thermal field are expressed as follows:

(1) $$\lambda \nabla^{2}T+Q=\rho C\frac{\partial T}{\partial t}$$

where *λ*, *ρ*, *C* denote the thermal conductivity, density and specific heat capacity, respectively; *Q* presents the volumetric loss due to copper loss and core loss; *T* is the temperature; *t* is the time variable; ∇ is the Hamiltonian operator.

The boundary condition is governed primarily by convective heat transfer between the transformer surface and the surrounding air:

(2) $$-\frac{\partial T}{\partial n}=h(T_{e}-T)$$

where *h* is the convective heat transfer coefficient; *T_e_* is the environmental temperature; and ***n*** is the external normal unit phasor.

As the temperature rises, the density of transformer oil changes, leading to oil circulation. This circulation, in turn, affects the temperature distribution, resulting in a thermal–fluid coupling process [25]. The governing equations for the oil flow are as follows:

(3) $$\left\{\begin{array}{l} \rho \frac{\partial v}{\partial t}+\rho (v\cdot \nabla )v=\nabla \times [-\rho I_{n}+K]+\rho g\\ \frac{\partial \rho }{\partial t}+\nabla \times (\rho v)=0\\ K=\mu (\nabla v+{\left(\nabla v\right)}^{T})-\frac{2}{3}\mu (\nabla \cdot v)I_{n} \end{array}\right.$$

where *ρ*, ***v*** represent the density and velocity of oil, respectively; ***I****_n_* denotes an n-order identity matrix; ***K*** is the stress tensor of viscous fluid; and ***g*** is the gravitational acceleration vector.

By solving Equations (1)–(3), we can obtain the temperature distribution of transform. These simulation results serve as data samples for training and validating the temperature prediction model. In this study, the thermal–fluid field equations are solved using the finite element method (FEM) in the COMSOL Multiphysics 6.3.

### 2.2. Simulation Model

**Figure 1:**
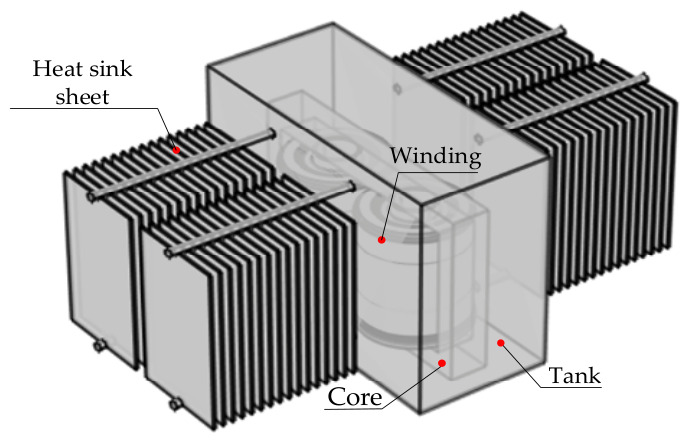
Simulation model for transformer temperature field.

In this study, a converter transformer model is used to simulate the temperature field. The model is a four-limb double-winding oil-immersed transformer, developed based on the D-800/35 scaled single-phase converter transformer prototype provided by Xi’an XD Transformer Co., Ltd. (Xi’an, China). The high-voltage winding is connected in parallel across two limbs, while the low-voltage winding is connected in series across the other two limbs. The physical simulation model and its main parameters are presented in Figure 1 and Table 1.

To improve simulation efficiency, components with minimal impact on the temperature field, such as the insulation bushings, oil conservator, and other auxiliary structures, are equivalently simplified in the simulation mode.

### 2.3. Simulation Results

**Figure 2:**
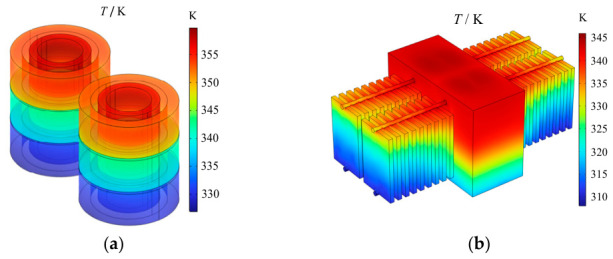
Temperature field simulation results of the transformer for (**a**) coil and (**b**) surface.

Based on the established physical and mathematical model of thermal–fluid coupling in the transformer, the finite-element simulation is conducted using COMSOL software. The electromagnetic–thermal coupling and fluid dynamics modules are employed to define the appropriate excitation conditions and material parameters are provided in Table 1. A convective heat transfer coefficient of *h* = 5 W/(m^2^·K), representing natural air convection, is applied as the boundary condition, and the ambient temperature is set to 290 K. The steady-state temperature distribution of the transformer under rated load conditions is shown in Figure 2.

The temperature distribution of the transformer windings and the overall body exhibits a typical gradient, with higher temperatures in the upper regions and lower temperatures in the lower regions. The hotspot temperature of the windings reaches approximately 87 °C.

## 3. Winding Temperature Prediction Based on XGBoost-LSTM

In practical engineering applications, direct measurement of internal winding temperature is challenging, whereas surface temperature monitoring is relatively easy to implement. The objective of the winding temperature prediction model is to establish a mathematical inverse mapping from surface temperature data to internal winding temperature. In this study, the XGBoost-LSTM algorithm is employed to achieve this goal.

### 3.1. Winding Temperature Prediction Model

**Figure 3:**
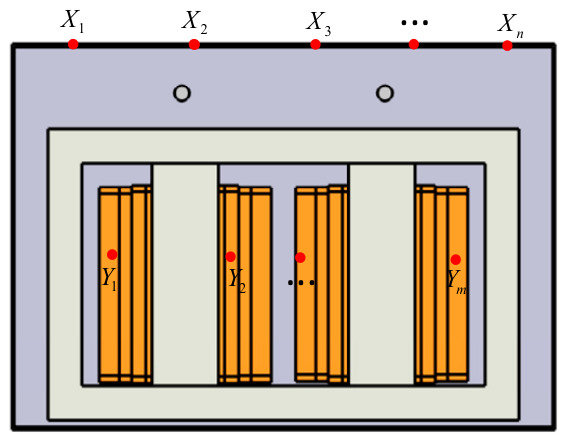
Schematic of internal and surface temperature points in the converter transformer.

To introduce the winding temperature prediction model, we build a 2D schematic diagram of the single-phase converter transformer in Figure 3 as a representative example. Let the measurable temperatures on the transformer surface be denoted as *X*_1_, *X*_2_, …, *X_n_*, and the internal temperature points to be predicted as *Y*_1_, *Y*_2_, …, *Y_m_*. The prediction model can therefore be expressed as:

(4) Y=f(X)

where *f* represents the inverse mapping operator, ***X*** = [*X*_1_, *X*_2_, …, *X_n_*]^T^ is the surface temperature vector, and ***Y*** = [*Y*_1_, *Y*_2_, …, *Y_m_*]^T^ is the internal winding temperature vector.

### 3.2. XGBoost-LSTM Algorithm for the Prediction Model

The winding temperature prediction model is implemented using a combination of XGBoost [26] and LSTM algorithms [27]. The implementation principle is as follows: although a large amount of surface temperature data can be collected, not all surface points exhibit strong correlation with internal winding temperatures. Therefore, XGBoost is first used as a feature selection algorithm to identify surface temperature points that are significantly correlated with internal winding temperatures. These selected surface temperatures serve as inputs for predicting the internal winding temperature. XGBoost integrates regression trees with gradient boosting, and its global objective function is defined as:

(5) $$obj=\sum_{i=1}^{N}L(Y_{i},\hat{Y_{i}})+\sum_{k=1}^{K}\Omega \left(f_{k}\right)$$

where *L* represents the loss function, *Ω* represents the complexity of the tree, *N* is the total number of input temperature samples, *K* is the total number of trees in the model, *f_k_* is the *k*-*th* tree in the training model, $Y_{i}={\left[Y_{1},Y_{2},\ldots ,Y_{m}\right]}_{i}$ and $Y_{i}={\left[{\hat{Y}}_{1},{\hat{Y}}_{2},\ldots ,{\hat{Y}}_{m}\right]}_{i}$ are the label values and predicted values of temperature samples for the *i*-*th* sample, respectively.

To minimize the objective function (5), XGBoost employs a greedy strategy, in which the residuals from the current base learner are used to train the next learner, thereby progressively optimizing the objective function during training. In the *k-th* iteration, the objective function can be expressed as:

(6) $${obj}^{\left(k\right)}=\sum_{i=1}^{N}L(Y_{i},F_{k-1}(X_{i})+f_{k}(X_{i}))+\Omega \left(f_{k}\right)$$

where $X_{i}={\left[x_{1},x_{2},\ldots ,x_{n}\right]}_{i}$ is the surface temperature for the *i*-*th* sample; $F_{k-1}(X_{i})$ and $f_{k}(X_{i})$ represent the prediction values obtained through *k*-1 tree and *k*-*th* tree, respectively.

Furthermore, we perform second-order Taylor expansion on formula (6) and obtain:

(7) $${Obj}^{\left(k\right)}≃\sum_{i=1}^{N}[L(Y_{i},F_{k-1}(X_{i}))+g_{i}f_{k}(X_{i})+\frac{1}{2}h_{i}f_{k}^{2}(x_{i})]+\Omega (f_{k})$$

where $g_{i}$, $h_{i}$ are the first-order and second-order gradients, respectively.

In the XGBoost algorithm, gain is a key metric used to evaluate feature importance. It quantifies the improvement in the loss function resulting from a tree node split. A higher gain indicates that the corresponding feature contributes more significantly to reducing prediction error, thereby enhancing the model’s predictive accuracy and generalization during training. The gain is calculated as:

(8) $$gain=\frac{1}{2}[\frac{{\left(\sum_{i\in I_{L}}g_{i}\right)}^{2}}{\sum_{i\in I_{L}}h_{i}+\lambda }+\frac{{\left(\sum_{i\in I_{R}}g_{i}\right)}^{2}}{\sum_{i\in I_{R}}h_{i}+\lambda }-\frac{{\left(\sum_{i\in I}g_{i}\right)}^{2}}{\sum_{i\in I}h_{i}+\lambda }]-\gamma$$

where *I*_L_ and *I*_R_ represent the left and right node samples after tree splitting; $I=I_{L}\cup I_{R}$, *λ* and *μ* are regularization coefficients.

**Figure 4:**
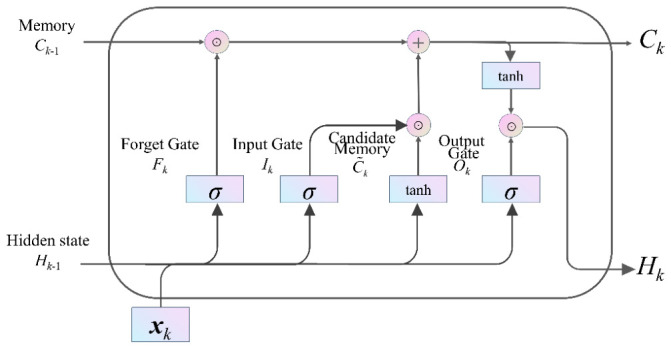
Principle diagram of a LSTM cell.

After selecting surface temperature points using XGBoost, the transformer temperature prediction model is trained using LSTM recurrent neural networks (RNNs), considering the sequential nature of temperature variations. LSTM is a specialized form of RNN designed to address the vanishing gradient problem commonly encountered in long-sequence modeling. With its unique gating mechanism, LSTM enables effective modeling of long-term dependencies, dynamic information filtering, and stable gradient propagation. These characteristics make LSTM particularly well-suited for transformer temperature prediction, which involves time-dependent, periodic, and occasionally abrupt fluctuations in state parameters. The schematic structure of an LSTM cell unit is illustrated in Figure 4:

The mathematical principle of cell unit gating mechanism of LSTM training process is as follows:

Forget gate $F_{k}$:

(9) $$F_{k}=\sigma (W_{f}\times [H_{k-1},x_{k}]+b_{f})$$

where $F_{k}$ determines which historical information should be retained or forgotten. *W_f_* and *b_f_* represent the weight matrix and bias vector of the forget gate, and σ is the sigmoid activation function.

Input gate *I_k_*, and candidate cell status ${\tilde{C}}_{k}$:

(10) $$\begin{array}{l} I_{k}=\sigma (W_{i}\times [H_{k-1},x_{k}]+b_{i})\\ {\tilde{C}}_{k}=\tan h(W_{C}\times [H_{k-1},x_{k}]+b_{C}) \end{array}$$

As expressed in Equation (10), *I_k_* and ${\tilde{C}}_{k}$ control the incorporation of new information into the cell. Here, *W_i_*, *W_C_* and *b_i_*, *b_C_* are the weight matrices and bias vectors for the input gate and candidate state, respectively.

Output gate *O_k_*:

(11) $$\begin{array}{l} O_{k}=\sigma (W_{O}\times [H_{k-1},x_{k}]+b_{O})\\ H_{k}=O_{k}⊙\tan hC_{k} \end{array}$$

where *O_k_* and *H_k_* are calculated to determine the output content of the current moment. *W_O_* and *b_O_* are the weight matrix and bias vector of the output gate.

### 3.3. Evaluation Indicators and Implementation Flow of the Prediction Model

To assess the accuracy of the temperature prediction model, the errors between predicted and true temperature values are evaluated using several metrics, including Mean Squared Error (*MSE*), Mean Absolute Error (*MAE*), and coefficient of determination *R*^2^. They are calculated as:

(12) $$\left\{\begin{array}{l} MSE=\frac{1}{n}\sum_{i=1}^{n}{\left(y_{i}-\;{\hat{y}}_{i}\right)}^{2}\\ MAE=\frac{1}{n}\sum_{i=1}^{n}\left|y_{i}-\;{\hat{y}}_{i}\right|\\ R^{2}=1-\frac{\sum_{i=1}^{n}{\left(y_{i}-{\hat{y}}_{i}\right)}^{2}}{\sum_{i=1}^{n}{\left(y_{i}-\;\bar{y}\right)}^{2}} \end{array}\right.$$

According to the definitions of the evaluation indicators, smaller values of *MSE* and *MAE* and an *R*^2^ value closer to 1 indicate higher model accuracy.

**Figure 5:**
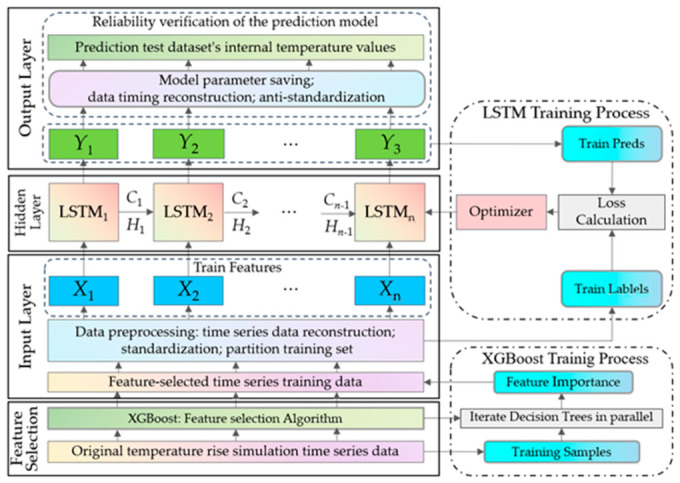
Flowchart of the temperature field prediction model development.

To support the training of the prediction model, multi-condition temperature field sample data were generated through FEM simulations of coupled electromagnetic–thermal–fluid multiphysics fields. By integrating the algorithms described above, the overall implementation process is illustrated in the flowchart shown in Figure 5.

The functions of each layer in the proposed model are as follows:

1. Feature selection layer: This layer takes the original temperature data generated by the simulation model as input and evaluates the feature importance of each surface measurement point using the XGBoost algorithm. To balance training cost and model accuracy, only the data corresponding to the most important features are retained and passed to the input layer.
2. Input layer: The input layer normalizes the selected features and transforms the data into a rolling time series format suitable for LSTM training, preparing it for processing by the hidden layer.
3. Hidden layer: The hidden layer adopts the LSTM time series network structure. The LSTM model is trained by iteratively optimizing the loss function using the training set data, allowing it to capture temporal dependencies in the input sequence.
4. Output layer: The output layer stores the trained network structure and internal weights. In this layer, inverse normalization and data reconstruction are performed on the model outputs. Reliability validation is also conducted to assess the accuracy of the predictions.

By following the above process to construct the XGBoost-LSTM prediction model, the internal winding temperature of the transformer can be effectively estimated using surface temperature measurements under corresponding operating conditions.

## 4. Reliability and Accuracy Validation on the Prediction Model

In this section, the reliability and accuracy of the proposed temperature prediction model are validated using both simulation data obtained from FEM simulations and experimental test data from the D-800/35 scaled converter transformer. First, the effectiveness of the XGBoost algorithm in evaluating the feature importance of surface temperature measurement points with respect to internal winding temperature is assessed. Based on this evaluation, the five most significant surface temperature points are selected as input features for the prediction model. The accuracy of the predicted internal winding temperatures is then validated through both simulation results and experimental measurements. And an engineering feasibility analysis was conducted for the proposed method.

### 4.1. Reliability of Surface Feature Temperature Point Selection

**Figure 6:**
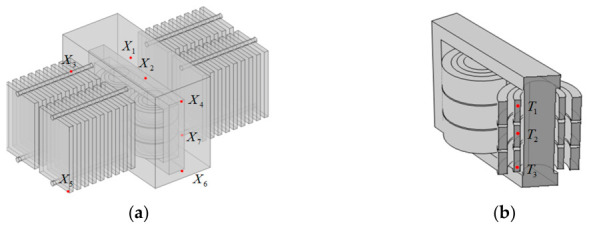
Schematic diagram of temperature point selection for (**a**) surface feature temperature measurement points; (**b**) internal point to be predicted.

**Figure 7:**
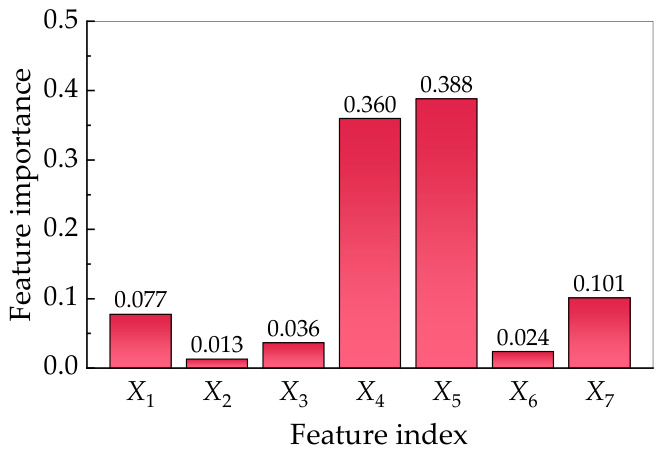
Screening results of different feature selection algorithms.

Surface feature temperature point selection is essential for optimizing the temperature sensor placement strategy. The physical model used for both simulation and experimental validation is consistent, as shown in Figure 6. Seven key surface temperature measurement points are initially selected along the main oil circulation path, where thermal gradients are expected to be most prominent. Additionally, three internal temperature points, located at 96% (top), 54% (middle), and 4% (bottom) of the axial height of the low-voltage winding, are chosen to evaluate the prediction accuracy. These internal locations correspond to positions where temperature sensors are installed in the experimental transformer. The spatial distribution of both surface and internal temperature points is illustrated in Figure 6. After training with the XGBoost algorithm, the feature importance of the surface temperature points, expressed as relative percentages, is presented in Figure 7.

As shown in Figure 7, there exists a complex nonlinear coupling relationship between surface and internal temperatures. This is reflected in the fact that all surface temperature points exhibit positive feature importance values, indicating that no input is redundant. Notably, the feature importance of *X*_4_ and *X*_5_ is significantly higher than that of the others, suggesting a stronger correlation with internal winding temperature. In contrast, points *X*_6_ and *X*_2_ exhibit comparatively lower feature importance. The feature importance indicated that *X*_4_ and *X*_5_ contribute more substantially to the prediction of internal winding temperature.

**Figure 8:**
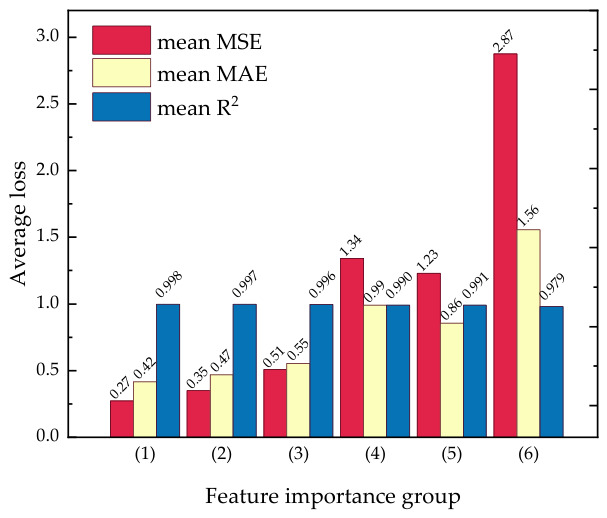
Comparison of prediction performance for different surface temperature point combinations based on XGBoost feature selection.

To verify the reliability of surface feature point selection using the XGBoost algorithm, we evaluated the impact of different surface temperature point groups that each with varying feature importance, on the accuracy of internal winding temperature prediction. The selected groups and their descriptions are provided in Table 2. Simulation results and experimental test data were used as the training and validation datasets, respectively, during the LSTM model training process. Each group was trained five times, and the evaluation indicators defined in Equation (12) were averaged to assess prediction accuracy. The results are presented in Figure 8.

As shown in Figure 8, the *MSE* and *MAE* errors in groups (1), (2), and (3) are relatively low, all of which include at least the top three most important surface temperature points. When these top three points are excluded from the input set, the prediction errors increase significantly. The indicator *R*^2^ follows a similar trend, though its sensitivity to feature group variation is less pronounced than that of *MSE* and *MAE*. A comparison between groups (4) and (5) reveals that using only the least important three points or just the single most important point yields similar prediction accuracy. However, the reduced number of measurement points in group (5) leads to larger error fluctuations, indicating greater instability in prediction performance. In summary, it can be seen that as the importance of the feature combination decreases, the volatility of the prediction effect increases.

Overall, the results demonstrate that as the combined feature importance of the selected surface temperature points decreases, the volatility of prediction accuracy increases. These findings validate the reliability of XGBoost-based feature importance screening for transformer surface temperature points. In practical applications, a balance must be struck between the number of sensors deployed, the model training time, and the accuracy of internal temperature prediction. To maintain both accuracy and computational efficiency, this study selects group (2) which includes the top five most important surface temperature points, for subsequent prediction accuracy validation.

### 4.2. Accuracy of the Winding Temperature Prediction

#### 4.2.1. Validation Using Simulation Data

To evaluate the accuracy and traceability of the XGBoost-LSTM prediction model for internal winding temperature estimation, simulation data under different operating conditions were first used for validation. To generate a diverse set of temperature samples, three operating parameters were varied in the COMSOL thermal–fluid field simulation: the transformer load rate, the convective heat transfer coefficient of air, and the operating duration. Specifically, the load rate was set to 0.85, 0.90, 0.95, 1.00 and 1.05; the convective heat transfer coefficient of air was set to 5, 6, 7, 8, 9, and 10 W/(m^2^·K); and the operating duration was set to 5, 6, 7, 8, and 9 h. By combining these parameters, 150 simulation cases were obtained in total.

**Figure 9:**
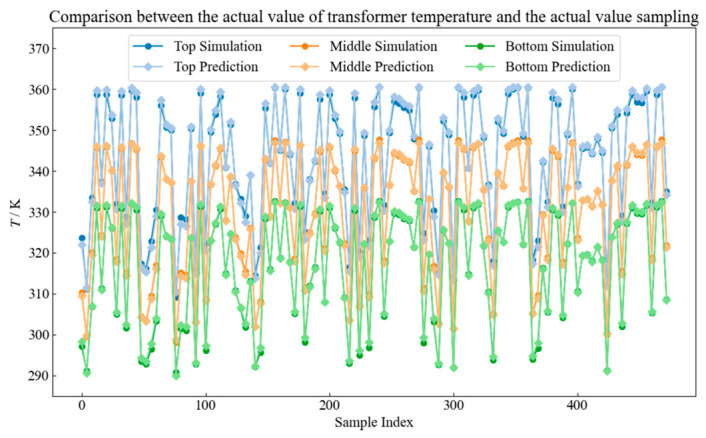
Accuracy verification results for temperature prediction model based on simulation data.

For each simulation case, the surface temperatures at the selected external feature measurement points were extracted and used as the input variables of the trained XGBoost-LSTM model. The model then predicted the internal winding temperatures at the top, middle, and bottom positions. These predicted values were compared with the corresponding internal winding temperatures obtained from the COMSOL simulation. Therefore, each simulation case produced three comparison results, corresponding to the top, middle, and bottom winding positions, and a total of 504 comparison data points were obtained. The prediction results and their comparison with the COMSOL reference values are shown in Figure 9.

As shown in Figure 9, the internal winding temperatures predicted by the XGBoost-LSTM model under the 150 simulated operating conditions are in good agreement with the COMSOL reference values. For the 450 comparison data points, the prediction errors remain small at all tested winding positions, with maximum errors of 1.2 K, 1.3 K, and 1.5 K for the top, middle, and bottom winding positions, respectively. These results indicate that the proposed model can accurately estimate the internal winding temperature from the selected external surface-temperature inputs under different simulated thermal loading conditions.

#### 4.2.2. Validation Using Experimental Test Data

To further validate the accuracy and traceability of the XGBoost-LSTM prediction model for internal temperature prediction, a temperature rise test was conducted on the D-800/35 scaled converter transformer prototype. The temperature measurement system mainly consisted of fiber Bragg grating (FBG) temperature sensors and an 8-channel high-precision grating fiber optic intelligent demodulator (FBG-1-3200) provided by Herch Opto Electronic Technology Co., Ltd. (Xi’an, China). The FBG temperature sensors were inserted through the flange plate and arranged at the predetermined internal winding measurement positions of the transformer. The GI-100/140P optical fiber was used in the system. During the test, the temperature-related optical signals were demodulated by the FBG-1-3200 and converted into temperature data. The measured temperature values were displayed in real time on the temperature-rise monitoring platform and recorded by a computer. The temperature measurement resolution of this solution is 0.1 K, and the measurement accuracy is ≤±1 K after calibration. Therefore, the optical fiber measurement system is capable of capturing the transient temperature variation during the temperature rise test.

**Figure 10:**
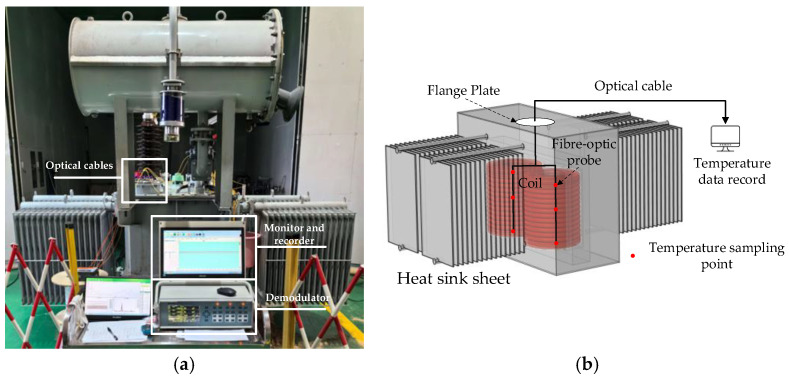
Test platform for transformer temperature rise and its temperature measurement system (**a**) photo of test platform and (**b**) wiring diagram of temperature measurement system.

Experimental temperature rise data were collected and used for model verification. In the experiment, the locations of the external surface temperature measurement points and the internal winding temperature sensors were kept consistent with those defined in the simulation model, so that the simulated data, measured data, and predicted data could be compared under the same spatial measurement configuration. The temperature rise test platform, and the layout of the optical fiber temperature sensors are shown in Figure 10.

According to the standard IEC 60076-2 [28], the temperature rise test is considered to have reached thermal stability when the rate of change in top-oil temperature is less than 1 K per hour. In this experiment, temperature data were recorded every 30 s over a duration of 480 min with the ambient temperature remaining around 290 K with ventilation. Therefore, the experimental dataset contains the complete transient temperature-rise process from the initial heating stage to the near-steady-state stage. The measured external surface temperatures at the selected points were used as the input variables of the XGBoost-LSTM prediction model, while the measured internal winding temperature obtained from the optical fiber sensor was used as the reference value for evaluating the prediction accuracy. Additionally, the transformer’s temperature field under the same operating conditions was simulated using COMSOL for comparative analysis.

The data-processing procedure for the experimental comparison was as follows. First, the real-time surface-temperature sequences collected from the selected external points were arranged in chronological order. Second, these surface-temperature sequences were input into the trained models to calculate the corresponding internal winding temperature at each sampling time. Finally, the predicted winding temperature was compared with the internal winding temperature measured by the optical fiber sensor and the winding temperature obtained from the COMSOL simulation.

**Figure 11:**
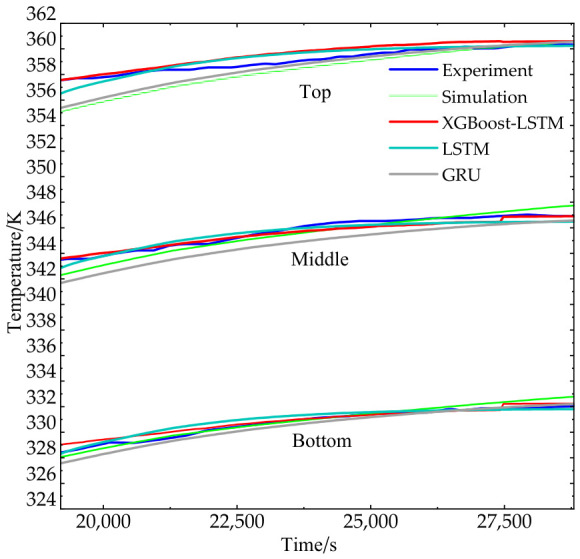
Comparison of temperature rise curves of internal points under experimental conditions.

**Figure 12:**
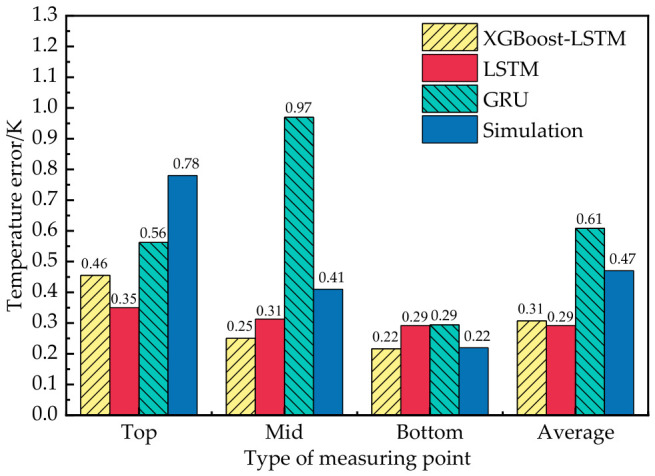
Comparison of average temperature error of each measuring point.

In the later stage of the experiment where the temperature approaches steady state, the temperature fluctuation is relatively small, the internal winding temperatures obtained from experimental measurements, COMSOL simulations, and predictions are compared in Figure 11. To further verify the effectiveness of the proposed XGBoost-LSTM method, additional comparative experiments were conducted using pure LSTM and Gated Recurrent Unit (GRU) models [19]. The errors of the simulation results and prediction results relative to the measured values were calculated separately, as shown in Figure 12.

Analysis of the results demonstrates that the XGBoost-LSTM prediction model achieves high accuracy in estimating the internal winding temperatures. The average prediction errors relative to the measured temperature rise data at the top, middle, and bottom points are approximately 0.46 K, 0.25 K, and 0.22 K, and the maximum errors (shown in Figure 11) in all sample data are approximately 0.83 K, 0.51 K, 0.62 K, respectively, indicating a great predictive performance.

Compared with physical measurement and FEM simulation, the proposed method offers a more efficient and practical approach, as it requires only matrix transformations and vector calculations with fixed model parameters to generate temperature predictions. This highlights the model’s potential for real-time application in transformer temperature monitoring.

And the results show that the prediction error of XGBoost-LSTM is close to that of pure LSTM and lower than that of GRU. This is because transformer temperature evolution is a slow thermal dynamic process with strong temporal continuity, and LSTM is capable of capturing long-term temporal dependencies effectively. Therefore, when sufficient surface-temperature information is used as input, pure LSTM can also achieve good prediction performance.

However, compared with pure LSTM, the proposed XGBoost-LSTM method provides an additional feature-selection mechanism before time-series modeling. By identifying the surface temperature points that are most strongly correlated with the internal winding temperature, XGBoost helps reduce redundant input variables and provides a more interpretable basis for temperature-measurement point selection. Therefore, the advantage of the proposed method is reflected not only in prediction accuracy, but also in measurement-point optimization and engineering applicability.

### 4.3. Engineering Feasibility Analysis

From an engineering perspective, the surface temperature points selected by XGBoost should not be regarded as arbitrary sensor-installation positions on the transformer body. In the experimental validation of this study, contact-type temperature sensors, such as fiber-optic temperature sensors, were used to obtain reliable surface temperature data. However, in actual transformer operation and maintenance, utilities may not permit the additional installation of contact-type sensors on operating transformers due to insulation safety, installation permission, equipment management, and maintenance-related constraints.

Therefore, in practical applications, the selected points should be interpreted as externally observable surface-temperature regions. The candidate measurement regions should first be limited to accessible and observable areas on the transformer tank surface, while avoiding positions near high-voltage bushings, obstructed structures, or locations that may interfere with routine inspection and maintenance. For these feasible regions, surface-temperature information can be acquired using non-contact temperature-measurement methods, such as infrared temperature probes or infrared thermal imagers. The XGBoost algorithm is then used to select the most informative temperature-observation regions from this engineering-feasible candidate set.

Moreover, the results in Table 2 and Figure 8 show that the model can still achieve relatively high prediction accuracy when part of the temperature-measurement information is removed, indicating a certain robustness to the absence of some measurement points. For surface regions that are difficult to measure in actual applications, these regions can be excluded from the candidate set or substituted by adjacent observable regions, and the prediction model can then be re-trained or fine-tuned using the remaining available temperature inputs.

In addition, the present study was validated on one scaled single-phase oil-immersed converter transformer. Although the proposed XGBoost-LSTM framework is expected to remain applicable in a broader sense, the learned feature importance and model parameters are transformer-dependent because thermal behavior is affected by transformer rating, structural design, cooling mode, and ambient conditions. For this reason, applying the method to a different transformer should involve re-selection of surface points and re-training or fine-tuning of model parameters using target-unit data. Future work will therefore focus on cross-model validation across transformers with different ratings and cooling modes, as well as on transfer-learning strategies for reducing the amount of required retraining data.

## 5. Conclusions

To address the challenge of directly measuring the internal winding temperature of transformers due to sensor installation limitations, this paper proposes a prediction method based on the XGBoost-LSTM algorithm, using surface temperature data as input. Using a 35 kV single-phase oil-immersed scaled converter transformer as a case study, a fluid–solid coupled thermal simulation model was established, and a D-800/35 scaled prototype was constructed for experimental validation. Both simulation and experimental results indicate that the proposed model achieves high prediction accuracy, with internal winding temperature errors of less than 1.5 K. The method enables rapid, real-time estimation of internal temperatures without the need for intrusive sensing, offering significant value for transformer temperature monitoring and fault diagnosis. For practical deployment, the selected surface temperature points should be determined within engineering-feasible and observable regions, while future work will further validate the proposed framework on transformers with different ratings, cooling modes, and ambient conditions.

## Figures and Tables

**Table 1:** Main parameters of the transformer.

Technical Parameter	Value	Technical Parameter	Value	Technical Parameter	Value
Frequency (Hz)	50	HV side (kV)	35	Heat sink	Flange Assemble
Capacity (kVA)	800	LV side (kV)	10.5	Heat sink structure	Annular
Cooling mode	ONAN	Connection mode	Ii0		
**Geometric parameter**				**Value**	
Core (mm)		Inside diameter		196	
		Outside diameter		840	
HV coil (mm)		Inside diameter		410	
		Outside diameter		533	
		Height		484	
LV coil (mm)		Inside diameter		44	
		Outside diameter		18	
		Height		90	
**Material parameter**			**Value**		
Density (kg/m^3^)			1055.05 − 0.58 × T − 6.4 × 10^−5^ × T^2^		
Specific Heat [J/(kg·K)]			−13,408.15 + 123.04 × T − 0.34 × T^2^ + 3.1 × 10^−4^ × T^3^		
Viscosity (Pa·s)			91.45 − 1.33 × T^1^ + 7.8 × 10^−3^ × T^2^ − 2.3 × 10^−5^ × T^3^ + 3.3 × 10^−8^ × T^4^		
Thermal Conductivity [W/(m·K)]			0.1343 − 8.05 × 10^−5^ × T		
Density (kg/m^3^)			8960		
Specific Heat [J/(kg·K)]			385		
Thermal Conductivity [W/(m·K)]			400		
Density (kg/m^3^)			7870		
Specific Heat [J/(kg·K)]			450		
Thermal Conductivity [W/(m·K)]			45		

**Table 2:** Groups of temperature points with different feature importance.

Group	Temperature Points	Feature Importance
(1)	*X*_1_, *X*_2_, *X*_3_, *X*_4_, *X*_5_, *X*_6_, *X*_7_	all
(2)	*X*_1_, *X*_3_, *X*_4_, *X*_5_, *X*_7_	top five
(3)	*X*_4_, *X*_5_, *X*_7_	top three
(4)	*X*_2_, *X*_3_, *X*_6_	last three
(5)	*X*_5_	top one
(6)	*X*_2_	last one

## Data Availability

The datasets presented in this study are not publicly available due to confidentiality requirements of the industrial project. Requests to access the datasets should be directed to the corresponding author.
